# Emulating the local Kuramoto model with an injection-locked photonic crystal laser array

**DOI:** 10.1038/s41598-021-86982-w

**Published:** 2021-04-21

**Authors:** Naotomo Takemura, Kenta Takata, Masato Takiguchi, Masaya Notomi

**Affiliations:** 1 Nanophotonics Center, NTT Corp., 3-1, Morinosato Wakamiya Atsugi, Kanagawa, 243-0198 Japan; 2 NTT Basic Research Laboratories, NTT Corp., 3-1, Morinosato Wakamiya Atsugi, Kanagawa, 243-0198 Japan

**Keywords:** Lasers, LEDs and light sources, Optics and photonics, Physics, Optical physics, Statistical physics, thermodynamics and nonlinear dynamics, Nanoscale devices

## Abstract

The Kuramoto model is a mathematical model for describing the collective synchronization phenomena of coupled oscillators. We theoretically demonstrate that an array of coupled photonic crystal lasers emulates the Kuramoto model with non-delayed nearest-neighbor coupling (the local Kuramoto model). Our novel strategy employs indirect coupling between lasers via additional cold cavities. By installing cold cavities between laser cavities, we avoid the strong coupling of lasers and realize ideal mutual injection-locking with effective non-delayed dissipative coupling. First, after discussing the limit cycle interpretation of laser oscillation, we demonstrate the synchronization of two indirectly coupled lasers by numerically simulating coupled-mode equations. Second, by performing a phase reduction analysis, we show that laser dynamics in the proposed device can be mapped to the local Kuramoto model. Finally, we briefly demonstrate that a chain of indirectly coupled photonic crystal lasers actually emulates the one-dimensional local Kuramoto chain. We also argue that our proposed structure, which consists of periodically aligned cold cavities and laser cavities, will best be realized by using state-of-the-art buried multiple quantum well photonic crystals.

## Introduction

Nowadays, the investigation of synergetic dynamics emerging from coupled oscillators is an interdisciplinary study intensively discussed in physics, mathematics, chemistry, biology, and neuroscience^1^. Collective phenomena in coupled oscillators were investigated for the first time by Kuramoto, who used a large set of fully-connected oscillators which is a mathematical model called the Kuramoto model^2,3^. In spite of the simplicity of the Kuramoto model, it comprises rich physics. For example, it exhibits a phase transition-like phenomenon from an incoherent state to a fully synchronized state when coupling strength reaches a threshold. Investigations of the Kuramoto model are not limited to theoretical ones. Actually, it is the simplest model for understanding various collective synchronization phenomena observed in nature, such as the collective synchronizations of neural oscillations and fireflies. Another actively studied direction is the emulation of the Kuramoto model with physical systems, for which the well-known example is the Josephson junction array^4–6^, though another promising approach is to use an array of coupled lasers^7–17^. In this paper, we employ the latter approach and focus on a nanophotonic device, which provides an attractive playground for studying dynamical systems, with which synchronization of limit cycle oscillations has been theoretically and experimentally investigated^18–23^.

We propose a novel nanophotonic device that emulates the Kuramoto model with non-delayed nearest-neighbor coupling^3,24–28^, which we call the local Kuramoto model. Our idea is inspired by pioneering studies on coupled photonic crystal (PhC) lasers^29–34^ and by the mutual injection locking technique in laser physics^16,35–40^. Different from the conventional injection-locking^41,42^, mutual injection-locking involves neither master nor slave lasers. Our proposed device employs PhC lasers indirectly coupled via additional cold cavities. We demonstrate that the cold cavities play a crucial role in avoiding strong coupling between lasers, which results in ideal mutual injection-locking and dramatically simplifies the phase dynamics of laser oscillations. Compared with the other systems, nanophotonic Kuramoto models can be very compact devices that operate even at room temperatures. Furthermore, using PhC lasers, we aim for an on-chip realization of the local Kuramoto model, which may have an application as a coherent high-power laser. Additionally, in contrast to delayed coupling due to optical paths in free-space injection-locking^11,16^, our on-chip local Kuramoto model can provide stable coupling without coupling delay thanks to the direct evanescent coupling. Actually, the realization of dissipative coupling without time delay will be very difficult without using our scheme.

First, as a starting point, we consider two coupled PhC lasers coupled via a cold cavity. For this purpose, we interpret laser oscillation as limit cycle oscillation and model it by the Stuart-Landau equation. With coupled-mode equations, we numerically demonstrate the synchronization (mutual injection-locking) of two lasers. Furthermore, we confirm that strong-coupling between the two lasers is actually prohibited by the presence of the additional cold cavity. Second, in the same way as in our previous paper^43^, we perform a phase reduction analysis to calculate the phase equations of motion for two indirectly coupled lasers^2,44,45^. The obtained phase equations of motion indicate that the phase dynamics of lasers indirectly coupled via cold cavities is equivalent to the local Kuramoto model. Finally, we demonstrate that a one-dimensional chain of indirectly coupled PhC lasers can emulate the one-dimensional local Kuramoto chain^46^.

We also argue that our proposed device can be realized best by using buried multiple quantum well (MQW) PhC cavities^47–50^, where MQWs are locally embedded in a PhC slab. With this state-of-the-art technology, laser and cold cavities can be periodically aligned on a PhC chip.

## Lasers as limit cycle oscillators

Here, we review limit cycle interpretation for laser oscillation. In general, using complex field $\alpha$ and carrier number *N*, single-mode laser dynamics are, in the nonrotating frame, described by the following rate equations:^51–53^

1 $$\begin{aligned} \dot{\alpha }= & {} -i\omega _c\alpha -\frac{1}{2}\gamma _c\alpha +\frac{1}{2}\beta \gamma _\Vert N\alpha \end{aligned}$$

2 $$\begin{aligned} \dot{N}= & {} -\gamma _\Vert N-\beta \gamma _\Vert N|\alpha |^2+P, \end{aligned}$$

where *P* is the pumping rate for carriers, and $\omega _c$ is the resonance frequency of the laser cavity. Decay rates $\gamma _c$ and $\gamma _\Vert$ are photon and carrier decay rates, respectively. Note that, in this paper, by employing the quantum optics convention, the electric field rotates as $\alpha (t)=\alpha (0)e^{-i\omega _ct}$, which is opposite to the rotation in conventional coupled-mode equations, [$\alpha (t)=\alpha (0)e^{i\omega _ct}$]. The coefficient $\beta$ represents the fraction of photons spontaneously emitted into a lasing mode, and it is called the spontaneous emission coupling coefficient^51^. For simplicity, we neglect the linewidth enhancement factor in the rate equations (1) and (2) in the main text. In Section 5 in the supplemental material, we discuss the effect of the linewidth enhancement factor on synchronization, which may be negligible in quantum-dot lasers but generally has non-negligible effects in semiconductor lasers. Here, it is worth noting that, in Eqs. (1) and (2), the terms $\frac{1}{2}\beta \gamma _\Vert N\alpha$ and $-\beta \gamma _\Vert N|\alpha |^2$ represent the stimulated emission, while there are no spontaneous emission terms. The effect of spontaneous emission will be included in the rate equations through a field noise term, if necessary. It is also important to note that Eqs. (1) and (2) hold only for a low $\beta (\ll 1)$, which is usually the case in most lasers. The rate equations (1) and (2) are known to exhibit Hopf bifurcation, which is equivalent to lasing, when the pump rate reaches a lasing threshold $P=P_{\mathrm{th}}=\gamma _c/\beta$.

In this paper, for further simplification, we consider the case where the photon lifetime is much longer than the carrier lifetime ($\gamma _c\ll \gamma _\Vert$), which is called the class-A condition^54^. With this assumption, we adiabatically eliminate the carrier degree of freedom as $\dot{N}=0$^55,56^. The adiabatic elimination of the carrier dynamics reduces the rate equations (1) and (2) to

3 $$\begin{aligned} \dot{\alpha }=-i\omega _c\alpha +\frac{1}{2}\gamma _c\varepsilon \alpha -\frac{1}{2}\beta \gamma _c|\alpha |^2\alpha . \end{aligned}$$

**Figure 1:**
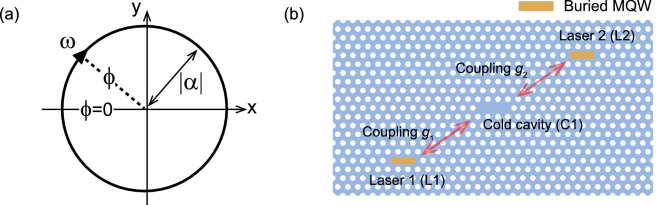
(**a**) Laser oscillation is interpreted as limit cycle oscillation in the nonrotating frame, where the laser frequency $\omega$ corresponds to the frequency of the limit cycle. With the amplitude $|\alpha |=\sqrt{\varepsilon /\beta }$ and phase $\phi =\omega t$ of the laser, we define the limit cycle orbit as $(x(\phi ),y((\phi )))=\sqrt{\varepsilon /\beta }(-\cos \phi ,\sin \phi )$, where *x* and *y* are the real $\mathrm{Re}[\alpha ]$ and imaginary parts $\mathrm{Im}[\alpha ]$ of the field, respectively. (**b**) Illustration of two PhC lasers (L1 and L2) indirectly coupled via a cold cavity (C1). The three cavities are evanescently coupled with coupling strengths $g_1$ and $g_2$.

Equation (3) is the well-known Stuart-Landau equation^2,57^, which is also called the Van der Pol equation^21,58^. Importantly, parameter $\varepsilon$ in Eq. (3) is the pump parameter defined as

4 $$\begin{aligned} \varepsilon \equiv \frac{P-P_{\mathrm{th}}}{P_{\mathrm{th}}}\ \ {\mathrm{with}}\ \ P_{\mathrm{th}}=\frac{\gamma _c}{\beta }, \end{aligned}$$

which indicates that the Hopf bifurcation (lasing) again occurs when $\varepsilon$ exceeds zero. Actually, when $\varepsilon >0$, the field amplitude $|\alpha |$ [see Fig. 1a] increases with an increase in the pump parameter as

5 $$\begin{aligned} |\alpha |=\sqrt{\frac{\varepsilon }{\beta }}\ \ \mathrm{for}\ \ \varepsilon \ge 0. \end{aligned}$$

Therefore, in Eq. (3), the linear $\gamma _c\varepsilon \alpha /2$ and nonlinear term $\beta \gamma _c|\alpha |^2\alpha /2$ can be interpreted as gain and gain saturation, respectively. Here, it is important to stress that the laser oscillation itself is interpreted as limit cycle oscillation, and thus the resonance frequency of the laser cavity $\omega _c$ is the oscillation frequency of the limit cycle. As limit cycle oscillation emerges only in a nonlinear dissipative system with energy injection, lasing is achieved with the cavity decay, pumping, and gain saturation (nonlinearity).

Finally, we briefly comment on the effect of photon-carrier dynamics on synchronization properties, which will be important in real PhC cavity lasers. Since PhC cavity lasers are semiconductor lasers, their carrier lifetime is much longer than the photon lifetime (sometimes called class-B lasers^54^), and the relaxation oscillation appears around lasing threshold^59,60^. Therefore, in real PhC cavity lasers, the adiabatic elimination approximation of the carrier degree of freedom cannot be justified, and we need to directly simulate the rate equations (1) and (2). Fortunately, we found that Eqs. (1) and (2) quantitatively provide the same results as the Stuart-Landau equation as long as phase dynamics are concerned, which can also be confirmed with the phase equation of motion for the class-B rate equations. See Section 4 in the supplemental material.

## Synchronization of two lasers

### Coupled-mode equations

Now, we consider the device shown in Fig. 1b, where the two lasers (L1 and L2) are indirectly coupled via the cold cavity (C1). The corresponding coupled-mode equations of motion representing field dynamics are given by

6 $$\begin{aligned} \dot{\alpha }_1= & {} -i\omega _1\alpha _1+\frac{1}{2}\gamma _1\varepsilon _1\alpha _1-\frac{1}{2}\beta _1\gamma _1|\alpha _1|^2\alpha _1-ig_1E_1 \end{aligned}$$

7 $$\begin{aligned} \dot{E}_1= & {} -i\Omega _1E_1-\frac{1}{2}\Gamma _1E_1-ig_1\alpha _1-ig_2\alpha _2 \end{aligned}$$

8 $$\begin{aligned} \dot{\alpha }_2= & {} -i\omega _2\alpha _2+\frac{1}{2}\gamma _2\varepsilon _2\alpha _2-\frac{1}{2}\beta _2\gamma _2|\alpha _2|^2\alpha _2-ig_2E_1, \end{aligned}$$

where $\alpha _{1,2}$ and $E_1$ represent fields in the laser cavity and cold cavity, respectively. Additionally, $\omega _{1,2}$ and $\Omega _1$ respectively represent the resonance frequencies of the laser cavities (L1 and L2) and coldcavity (C1). Similarly, $\gamma _{1,2}$ and $\Gamma _1$ are the field decay rates of the laser- (L1,2) and cold cavity (C1), respectively. The parameter $\beta _{1,2}$ is the spontaneous emission coupling coefficient, while $\varepsilon _{1,2}$ is the pump parameter for laser L1 and L2. Finally, the two coupling strengths between the cavities are denoted by $g_1$ and $g_2$. For simplicity, in the rest of this paper, we use $\beta _1=\beta _2=0.001$ and $\varepsilon _1=\varepsilon _2=1.0$, which is above the lasing threshold. Furthermore, we use the same values for the normalized decay rates of the laser cavity and cold cavity: $\Gamma _1=\gamma _2=\gamma _1\equiv 1$, where $\gamma _1$ is interpreted as a dimensionless parameter for numerical simulations.

To observe synchronization, we set the resonance frequencies of the two laser cavities as $\omega _2=\omega _1+\Delta \omega$ with $\Delta \omega =0.01\gamma _1$, where $\Delta \omega \equiv \omega _2-\omega _1$ is the frequency difference between the two lasers. For the cold cavity (C1), for simplicity, we use the same resonance frequency as L1: $\Omega _1=\omega _1$.

### Time evolutions

By showing field time evolutions described by the coupled-mode Eqs. (6)–(8), we demonstrate the synchronization of two lasers (mutual injection locking). Since the typical laser frequency, which is on the order of hundreds of terahertz, we perform the rotating-frame transformation for all fields, for example, as $\alpha _1e^{-i\omega _s t}\rightarrow \alpha _1$. With this rotating frame transformation, we shift the resonance frequencies of the cavities as $\omega _1^\prime \equiv \omega _1-\omega _s=1\gamma _1$, $\omega _2^\prime \equiv \omega _2-\omega _s=1.01\gamma _1$, and $\Omega _1^\prime \equiv \Omega _1-\omega _s=1\gamma _1$. Importantly, there is an arbitrariness in the absolute frequencies, and only the relative frequencies are important. Thus, the frequency of the rotating frame, $\omega _s$, is arbitrary, and only the relative values between $\omega _1$, $\omega _2$, and $\Omega _1$ matter.

**Figure 2:**
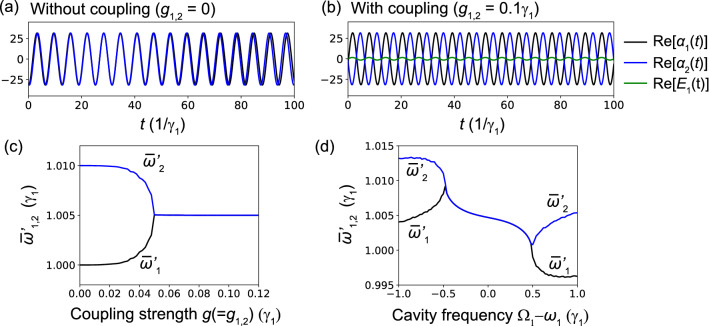
Simulations for two lasers [see Fig. 1b] coupled via a cold cavity. The simulated time evolutions of the real part of the field $\mathrm{Re}[\alpha _{1,2}(t)]$ without $g_{1,2}=0$ (**a**) and with coupling $g_{1,2}=0.1\gamma _1$ (b). Here, we used the shifted laser and cold cavity frequencies $\omega _1^\prime =1\gamma _1$, $\omega _2^\prime =1.01\gamma _1$, $\Omega _1^\prime =1\gamma _1$. In (**b**), we also show the time evolution of the real part of the field of the cold cavity $\mathrm{Re}[E_{1}(t)]$. (**c**) Mean frequency of the laser oscillation $\bar{\omega }_{1,2}$ as a function of coupled strength $g_{1,2}$. (**d**) Mean frequency $\bar{\omega }_{1,2}$ for fixed coupling strength ($g_{1,2}=0.1\gamma _1$) but as a function of the resonance frequency of the cold cavity $\Omega _1$, which tunes the effective coupling strength between the two lasers.

First, Fig. 2a shows the time evolutions of the real parts of the fields $\mathrm{Re}[\alpha _1(t)]$ (black) and $\mathrm{Re}[\alpha _2(t)]$ (blue) for the lasers L1 and L2, respectively, without coupling between cavities $g_1=g_2=0$. Without coupling between the cavities, there is no photon in cold-cavity C3, and thus $E_1(t)=0$. As we expect, in Fig. 2a, the fields in laser L1 and L2 oscillate with their own frequencies: $\omega _1^\prime =1\gamma _1$ and $\omega _2^\prime =1.01\gamma _1$. Second, we introduce coupling between the cavities as $g_1=g_2=0.1\gamma _1$ in Fig. 2b, where the green curve is the time evolution of the real part of the field in cold-cavity C1 $\mathrm{Re}[E_1]$. Figure 2b indicates that the two indirectly coupled laser oscillations exhibit synchronization (mutual injection locking), which is the main result of this paper. Furthermore, the synchronization phase is anti-phase, which is called anti-phase synchronization. Importantly, thanks to cold-cavity C3, normal-mode splitting associated with strong coupling between the two lasers is prohibited, which is confirmed from the fact that the frequency of the synchronized oscillations does not depend on the initial states of two lasers (not shown). In fact, when the system is in the strong-coupling regime, depending on the initial states of two lasers, for example, they form a “bonding” or “anti-bonding” mode, and their frequencies become lower or higher than the original oscillation frequencies^43^. Importantly, no matter how weak the coupling is, directly coupled lasers exhibit normal-mode splitting because they have no decay (gain). Note that, for Eqs. (6)–(8), anti-phase synchronization always occurs for any initial state, while if the signs of the two couplings are opposite such as $g_2=-g_1$, in-phase synchronization always occurs (not shown) The sign of a coupling constant depends on the overlap integral of cavity fields and may vary depending on the distance between cavities. In the device design in this paper, since all the distances between cavities are designed to be equal, all the signs of coupling constants can be assumed to be the same. In any case, the property that a synchronization phase does not depend on initial phases of lasers is of importance because the initial phase of PhC lasers cannot be controlled experimentally.

### Synchronization tree

In Fig. 2c, we show the mean frequencies of the two laser oscillations $\bar{\omega }_{1}^\prime$ and $\bar{\omega }_{2}^\prime$ as a function of the coupling between cavities $g_{1,2}$. Since, in general, limit cycle oscillations are quasi-periodic when coupling strength is lower than the critical strength of synchronization, we need to use their mean frequencies obtained with peak detection. Figure 2c clearly indicates that the mean frequencies symmetrically approach each other with an increase in the coupling strength *g* and that they merge as $\bar{\omega }_1^\prime =\bar{\omega }_2^\prime =1.005\gamma _1$ at the critical strength $g=0.05\gamma _1$. In fact, the frequency $\bar{\omega }_{1,2}^\prime =1.005\gamma _1$ is the mean frequency of $\omega _1^\prime =1\gamma _1$ and $\omega _2^\prime =1.01\gamma _1$ without coupling. Note that the synchronization tree shown in Fig. 2c is approximately symmetric for $\bar{\omega }_{1}^\prime$ and $\bar{\omega }_{2}^\prime$, which is because the parameters are almost the same for L1 and L2.

Furthermore, in Fig. 2d, we plot the mean frequency $\bar{\omega }_{1,2}^\prime$ as a function of the resonance frequency of the cold cavity $\Omega _1$, where the coupling strengths are fixed as $g_1=g_2=0.07\gamma _1$ while $\Omega _1$ is swept from $\omega _1-1\gamma _1$ to $\omega _1+1\gamma _1$. Figure 2d indicates that the effective coupling strengths between the cavities can be tuned by changing the resonance frequency of the cold cavity $\Omega _1$. Intuitively, as the cold-cavity’s frequency deviates from the resonance frequencies of the two lasers, the effective coupling strengths decrease. In PhC cavities, the tuning of cavity coupling strength, which is determined by the distance between cavities, is almost impossible. Meanwhile the tuning of the cold cavity’s resonance frequency is technically available with the carrier-injection^61,62^ or thermo-optic techniques^63,64^, and thus the synchronization tree shown in Fig. 2d could be measured.

## Phase equations of motion

In this section, as we did in Ref.^43^, by performing the phase reduction analysis^2,65^ for Eqs. (6)–(8), we attempt to obtain phase equations of motion. In our case, the phase of limit cycle oscillation is nothing else but the phase of a laser $\phi$ as illustrated in Fig. 1a, and thus the interpretation of corresponding phase equations of motion is also straightforward. Furthermore, we show that the determination of phase equations of motion is of importance in terms of mapping our model to the local Kuramoto model. The price to pay for obtaining phase equations of motion is the adiabatic elimination of the field in cold-cavity C1, which is required to transform the indirectly coupled system to a directly coupled model with dissipative coupling.

### Adiabatic elimination approximation

The adiabatic elimination of the cold-cavity field degree of freedom $\dot{E}_1=0$ requires that field $E_1$ rapidly decays compared with the laser field $\alpha _{1,2}$, and thus $E_1$ adiabatically follows $\alpha _{1}$ and $\alpha _{2}$. The time-scale of a variable is generally characterized by its decay rate. Therefore, the conventional adiabatic elimination of field $E_1$ requires that the decay rate $\Gamma _1$ must be larger than the decay rates of $\alpha _1$ and $\alpha _2$, as shown in “Lasers as limit cycle oscillators” section, which is not the case, for example, when we consider $\Gamma _1=\gamma _{1,2}$ as in Fig. 2. However, importantly, the time scale of the laser field $\alpha _{1,2}$ is not characterized solely by $\gamma _{1,2}$. Now, it is important to define the effective decay rates for $\alpha _1$, $\alpha _2$, and $E_1$, including both oscillation frequencies and pump parameters, as $\lambda _1\equiv -\gamma _1\epsilon _1/2+i\omega _1$, $\lambda _2\equiv -\gamma _2\epsilon _2/2+i\omega _1$, and $\Lambda _1\equiv \Gamma _1/2+i\Omega _1$, respectively. Here, the oscillation frequencies $\omega _1$, $\omega _2$, and $\Omega _1$ are the imaginary parts of the effective decay rates. First, as pointed out by Haken^56^, to compare the time scales of the variables, in the effective decay rates, the imaginary parts must be negligible compared to the real parts: $\mathrm{Im}[\lambda _{1,2}]\ll \mathrm{Re}[\lambda _{1,2}]$ and $\mathrm{Im}[\Lambda _{1}]\ll \mathrm{Re}[\Lambda _{1}]$. Even though the cavity resonance frequencies $\omega _{1,2}$ and $\Omega _1$ are always much higher than the terms $\gamma _{1,2}\epsilon _{1,2}/2$ and $\Gamma _{1}/2$, if all the resonance frequencies of the cavities have similar values $\omega _1\simeq \omega _2\simeq \Omega _1$, the imaginary parts in the effective decay rates become negligible in a rotating frame with the frequency of $\Omega _1$. Second, by comparing the real parts of the effective decay rates $\mathrm{Re}[\lambda _{1,2}]$ and $\mathrm{Re}[\Lambda _{1}]$, we find that the sign of $\mathrm{Re}[\Lambda _{1}]$ is always positive, while the sign of $\mathrm{Re}[\lambda _{1,2}]$ can be negative due to gain when the pump power is above the threshold $\varepsilon _{1,2}\ge 0$. According to Ref.^56,66^, when $\mathrm{Re}[\Lambda _{1}]>0$ and $\mathrm{Re}[\lambda _{1,2}]\le 0$, the field $E_1$ is a “stable” mode that rapidly decays, while the laser fields $\alpha _1$ and $\alpha _2$ are unstable modes that do not decay but govern the slow dynamics of the system, which allows putting $\dot{E}_1=0$ (adiabatic elimination). In fact, the unstable mode $\alpha _{1,2}$ “enslaves” the stable mode $E_1$ and plays a role as an “order parameter” (the slaving principle^56,66^).

Now, setting $\dot{E}_1=0$ for Eq. (7), we eliminate the cold-cavity field degree of freedom as

9 $$\begin{aligned} E_1=-i\frac{2}{\Gamma _1}(g_1\alpha _1+g_2\alpha _2). \end{aligned}$$

By substituting Eq. (9) into Eqs. (6) and (8), we obtain approximated equations of motion:

10 $$\begin{aligned} \dot{\alpha }_1= & {} -i\omega _1\alpha _1+\left[ \frac{1}{2}\gamma _1\varepsilon _1-\frac{2g_1^2}{\Gamma _1}\right] \alpha _1-\frac{1}{2}\beta _1\gamma _1|\alpha _1|^2\alpha _1-\frac{2g_1g_2}{\Gamma _1}\alpha _2 \end{aligned}$$

11 $$\begin{aligned} \dot{\alpha }_2= & {} -i\omega _2\alpha _2+\left[ \frac{1}{2}\gamma _2\varepsilon _2-\frac{2g_2^2}{\Gamma _1}\right] \alpha _2-\frac{1}{2}\beta _2\gamma _2|\alpha _2|^2\alpha _2-\frac{2g_1g_2}{\Gamma _1}\alpha _1. \end{aligned}$$

**Figure 3:**
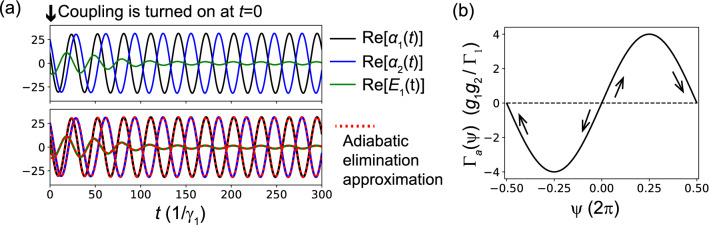
(**a**) Time evolution of the fields $\mathrm{Re}[\alpha _{1,2}(t)]$ and $\mathrm{Re}[E(t)_{1}]$, but coupling ($g_{1,2}=0.1\gamma _1$) is turned on at $t=0$, which represents synchronization dynamics. The upper panel shows the time evolutions of the fields calculated with the original coupled-mode equations. In the lower panel, the time evolutions of the fields calculated with the adiabatic elimination approximation are shown as red dashed lines with the original plots. The shifted frequencies of the laser and cold cavities are $\omega _1^\prime =0.2\gamma _1$, $\omega _2^\prime =0.21\gamma _1$, and $\Omega _1^\prime =0.2\gamma _1$. (**b**) Anti-symmetric part of the phase coupling function $\Gamma _a(\psi )$ given by Eq. (16), where $\psi$ is the phase difference between the two laser phases defined as $\psi \equiv \phi _2-\phi _1$.

To confirm the validity of this adiabatic elimination approximation, in Fig. 3a, we show synchronization dynamics calculated both with the original equations of motion (6)–(8) and approximated equations (10) and (11). In Fig. 3a, coupling with $g_{1,2}=0.1\gamma _1$ is switched on at $t=0$ for uncoupled steady-state laser oscillations, and thus the time evolutions of fields represent synchronization dynamics from the unsynchronized to synchronized state. The upper panel in Fig. 3a shows only the synchronization dynamics calculated with the original equations of motion (6)–(8). Meanwhile, in the lower panel, synchronizations calculated with the original equations of motion (solid lines) overlap those calculated with the approximated equations of motion (dashed lines), which clearly indicates that two time evolutions are almost indistinguishable and that the adiabatic elimination approximation is surprisingly good. Note that, to clearly show the synchronization dynamics in Fig. 3a, we used shifted frequencies $\omega _1^\prime =0.2\gamma _1$, $\omega _2^\prime =\omega _1^\prime +\Delta \omega =0.21\gamma _1$ and $\Omega _1^\prime =\omega _1^\prime =0.2\gamma _1$, which are lower than those Fig. 1. As we commented in “Coupled-mode equations” section, these shifts of the resonance frequencies do not change the physics, because only the relative relationship between the resonance frequencies is important. Since the field in the cold cavity was adiabatically eliminated, Eqs. (10) and (11) represent directly coupled lasers. Furthermore, in Eqs. (10) and (11), the effective couplings represented by $-(2g_1g_2/\Gamma _1)\alpha _2$ and $-(2g_1g_2/\Gamma _1)\alpha _1$ are non-energy-conserving dissipative couplings, which intuitively explains why normal-mode splitting does not appear in our model. Additionally, in Eqs. (10) and (11), the effective dissipative coupling does not have the time delay.

Finally, we comment on synchronization with a large coupling strength. We found that Eqs. (10) and (11) fail to reproduce synchronization dynamics when $g_{1,2}\ge \gamma _{1,2},\Gamma _1$, which is because the adiabatic elimination approximation cannot describe coherent intensity oscillation between cavities associated with this parameter region [see Section 2 in the Supplemental Material (SM)]. Therefore, the complete conditions required for the adiabatic elimination approximation are

12 $$\begin{aligned} \omega _1\simeq \omega _2\simeq \Omega _1\ \mathrm{and}\ g_{1,2}<\gamma _{1,2},\Gamma _1. \end{aligned}$$

Here, it is also important to stress that, although the adiabatic elimination fails to describe synchronization dynamics, even when $g_{1,2}\ge \gamma _{1,2},\Gamma _1$, stable synchronization itself can occur and the adiabatic elimination approximation well reproduces the steady-state synchronized oscillations (see Section 2 in the SM). Furthermore, even when the coupling is extremely strong, for example, $g_{1,2}=10\gamma _1$, we can observe stable synchronization, where no normal-mode splitting is present (not shown). This insensitivity to coupling strength will be advantageous in terms of real device designs, because adjusting the value of weak coupling strength is technically difficult^43^. Furthermore, if coupling is sufficiently strong, we may prove synchronization from spectral shapes, which is discussed again in “Discussion” section.

### Phase reduction analysis

Now, we perform the phase reduction analysis for equations of motion (10) and (11), which were obtained with the adiabatic elimination approximation. Here, we make use of the consequence of the phase reduction theory without going into the theoretical detail, which is briefly provided in Section 1 in the SM (further details can be found in our recent paper^43^ and in Refs^2,44^). The objective of the phase reduction analysis is to obtain the phase equations of motion for the phases of the laser L1 ($\phi _{1}$) and L2 ($\phi _{2}$) represented as

13 $$\begin{aligned} \dot{\phi _1}= & {} -\omega _1+\Gamma _{12}(\phi _1-\phi _2) \end{aligned}$$

14 $$\begin{aligned} \dot{\phi _2}= & {} -\omega _2+\Gamma _{21}(\phi _2-\phi _1), \end{aligned}$$

where $\Gamma _{12}(\phi )$ and $\Gamma _{21}(\phi )$ are called the phase-coupling functions. For the approximated equations of motion (10) and (11), we found that $\Gamma _{12}(\phi )$ and $\Gamma _{21}(\phi )$ can be analytically calculated as

15 $$\begin{aligned} \Gamma _{12}(\theta )=\Gamma _{21}(\theta )=\frac{2g_1g_2}{\Gamma _1}\sin \theta \end{aligned}$$

Finally, the phase difference between the two lasers $\psi \equiv \phi _2-\phi _1$ follows the following simple equation of motion:

16 $$\begin{aligned} \dot{\psi }=-\Delta \omega +\Gamma _a(\psi )\ \ \mathrm{with}\ \ \Gamma _a(\psi )=\frac{4g_1g_2}{\Gamma _1}\sin \psi , \end{aligned}$$

where $\Delta \omega \equiv \omega _2-\omega _1$ is the frequency difference between the two lasers already defined in “Coupled-mode equations” section. Here, $\Gamma _a(\psi )\equiv \Gamma _{21}(\psi )-\Gamma _{12}(-\psi )$ is the anti-symmetric part of the phase coupling function $\Gamma _{21}(\psi )$, which is shown in Fig. 3b. For a negligible laser frequency difference $\Delta \omega \simeq 0$, since $\Gamma _a(\pi )=0$ and $\Gamma ^\prime _a(\pi )<0$ hold for Eq. (16), phase locking occurs at the phase $\psi =\phi _2-\phi _1=\pi$, which is anti-phase synchronization as expected from the simulations [see the arrows in Fig. 3b]. Meanwhile, since $\Gamma _a(0)=0$ and $\Gamma ^\prime _a(0)>0$ hold for $\phi =0$, the phase $\phi =0$ is an unstable fixed point. Of course, for a non-negligible frequency difference $\Delta \omega \ne 0$, the synchronization phase shifts from $\pi$. The phase equations of motion predict not only the synchronization phase but also the critical coupling strength of synchronization. For Eq. (16) to have a phase-locking solution, the condition $-4g_1g_2/\Gamma _1\le \Delta \omega \le 4g_1g_2/\Gamma _1$ must be satisfied. For the oscillation frequency difference $\Delta \omega =0.01\gamma _1$ and cold-cavity decay rate $\Gamma _1=1\gamma _1$, which are assumed in Fig. 2c, synchronization occurs when the coupling strengths reach $g_1=g_2=0.05\gamma _1$ [see Fig. 2c] because the above phase-locking condition is satisfied with these parameters as $4g_1g_2/\Gamma _1=0.01\gamma _1=\Delta \omega$.

Furthermore, the analytically calculated phase coupling functions in Eq. (15) are also of importance for mapping our model to the local Kuramoto model. In fact, the phase equations of motion are explicitly written as

17 $$\begin{aligned} \dot{\phi _1}= & {} -\omega _1+\tilde{g}_{12}\sin (\phi _1-\phi _2) \end{aligned}$$

18 $$\begin{aligned} \dot{\phi _2}= & {} -\omega _2+\tilde{g}_{21}\sin (\phi _2-\phi _1), \end{aligned}$$

where $\tilde{g}_{ij}\equiv 2g_ig_j/\Gamma _1$ ($\tilde{g}_{ij}=\tilde{g}_{ji}$) is the effective coupling strength. The phase equations of motion (17) and (18) are straightforwardly extended to a one-dimensional chain or two-dimensional array as

19 $$\begin{aligned} \dot{\phi _i}=-\omega _i+\sum _{j\in N_i}\tilde{g}_{ij}\sin (\phi _i-\phi _j), \end{aligned}$$

where $N_j$ represents the nearest neighbour sites of the *i*th site. Importantly, the coupled phase oscillator described by Eq. (19) is equivalent to the local Kuramoto model^3,25^. Note that, in the original Kuramoto model, the sign of the coupling is minus as $-\tilde{g}_{21}$, and thus in-phase synchronization occurs.

In conclusion, with the aide of the phase reduction theory, we proved that an array of lasers with cold-cavity-mediated coupling can emulate the nearest-neighbor coupled Kuramoto model (the local Kuramoto model). Note that, of course, the strict mapping of given coupled-mode equations to the local Kuramoto model (19) requires an adiabatic elimination condition similar to Eq. (12).

## Array configuration

**Figure 4:**
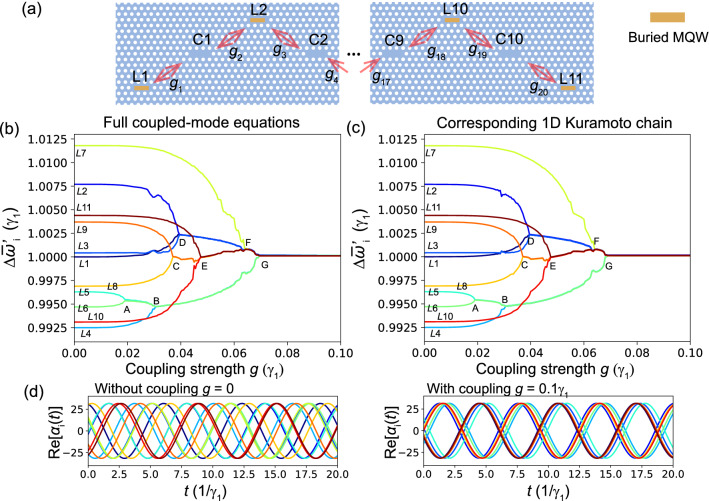
(**a**) Schematic of a chain of eleven indirectly coupled PhC lasers that emulates the Kuramoto chain. Indices L*i* and C*i* represent the *i*th laser and cold cavities, respectively. The shifted resonance frequencies of the laser cavities are $\omega _1^\prime =$1.0000, $\omega _2^\prime =$1.0077, $\omega _3^\prime =$1.0004, $\omega _4^\prime =$0.9925, $\omega _5^\prime =$0.9963, $\omega _6^\prime =$0.9947, $\omega _7^\prime =$1.0118, $\omega _8^\prime =$0.9969, $\omega _9^\prime =$1.0037, $\omega _{10}^\prime =$0.9931, and $\omega _{11}^\prime =$1.0044, where the units are $\gamma _1$. For the other parameters, we use $\beta _i=0.001$, $\varepsilon _i=1.0$, $\gamma _i\equiv 1$, $\Omega _i=1\gamma _1$ and $\Gamma _i=\gamma _1\equiv 1$ for all *i*. (**c**) Synchronization tree calculated with the local Kuramoto chain (Eq. (19)) corresponding to (**b**). (**b**) The mean oscillation frequencies of the eleven lasers $\bar{\omega }_i^\prime$ are shown as a function the coupling strength $g_i=g$ for all *i*. The synchronization points are denoted by A–G. (**d**) Time evolutions of the real parts of the fields in all the laser cavities without $g=0$ (left) and with coupling $g=0.1\gamma _1$ (right).

Although the investigation of rich physics emerging from coupled phase oscillators is beyond the scope of this paper, we briefly simulate a one-dimensional chain of indirectly coupled PhC lasers and demonstrate that our device can actually reproduce collective dynamics predicted for the one-dimensional local Kuramoto chain^46^. The chain of indirectly coupled PhC lasers is schematically illustrated in Fig. 4a, where eleven laser cavities and ten cold cavities are alternately aligned. Of course, the configuration of cavities to realize the local Kuramoto chain is not limited to that shown in Fig. 4a, and various configurations can be imagined. For a one-dimensional chain, in principle, even the periodic boundary condition may be implemented with a ring-like configuration. For simplicity, for all the laser cavities, we assume $\beta _i=0.001$, $\varepsilon _i=1.0$, and $\gamma _i\equiv 1$. Similarly, all the cold cavities have the same resonance frequencies and photon decay rate: $\Omega _i=1\gamma _1$ and $\Gamma _i=\gamma _1\equiv 1$ for all *i*. In Section 6 in the supplemental material, we demonstrate large-scale synchronization when the parameter values of all laser and cold cavities are slightly different. Furthermore, as in “Synchronization of two lasers” section, we assume that all the coupling constants have the same strengths: $g_{i}=g$ for all *i*. Meanwhile, the resonance frequencies of the eleven laser cavities are randomly distributed around a mean frequency $\bar{\omega }_i=1\gamma _1$ [for the actual values of $\omega _i$, please see the caption of Fig. 4]. Note that since all the laser and cold cavities have similar resonance frequencies and the coupling strengths are smaller than the cavity decay rates, an adiabatic elimination condition similar to Eq. (12) is satisfied, and thus corresponding simple phase equations of motion are expected to exist.

By directly simulating the full coupled-mode equations corresponding to the configuration shown in Fig. 4a, we calculated the mean frequencies of the laser oscillations as a function of the coupling strength *g* [see the synchronization tree in Fig. 4b]. As Fig. 4b indicates, with an increase in coupling strength *g*, synchronized clusters are gradually formed, and finally all clusters merge into a single fully synchronized cluster at $g\simeq 0.07\gamma _1$ [see G on Fig. 4b]. Similarly to Ref.^46^, when two [at A, B, C in Fig. 4b] or three [at D, E in Fig. 4b] adjacent oscillators (or clusters) have close oscillation frequencies, they form a new synchronized cluster with an increase in coupling strength. When adjacent clusters have largely different frequencies, while non-adjacent clusters have similar frequencies, the non-adjacent clusters form a synchronized cluster. In fact, the synchronization denoted by F in Fig. 4b consists of the non-adjacent oscillators (clusters) L1-3 and L7-11. Furthermore, in Fig. 4c, we show a synchronization tree calculated with the local Kuramoto chain (Eq. 19) corresponding to Fig. 4b. The fact that both synchronization trees have almost the same structures indicates that our proposed device will actually emulate the local Kuramoto model.

Finally, the time evolutions of the laser oscillations without ($g=0$) and with coupling ($g=0.1\gamma _1$) are shown in the left and right panels of Fig. 4d, respectively. When there is no coupling, as we expect, the laser oscillations are totally uncorrelated, while all the laser oscillations are fully synchronized with coupling $g=0.1\gamma _1$. Interestingly, in this fully synchronized state [see the right panel in Fig. 4d], the phases are opposite between the even and odd sites of the lasers oscillations. Therefore, even in the one-dimensional chain, a pair of adjacent laser oscillations exhibit anti-phase synchronization. Note that, in Fig. 4c,d, the “de-synchronization” discovered in^46^ was not observed, which may be due to the small number of oscillators or, more interestingly, could be associated with anti-phase synchronization. We also comment on the offsets of the synchronization phases in the fully-synchronized oscillations shown in the right panel of Fig. 4d, where the synchronization phases slightly differ depending on the pair of the synchronized oscillations. We found that, with a further increase in coupling strength, these offsets of the synchronization phases disappear and that all the pairs of synchronized oscillations become indistinguishable.

## Discussion

Here, we discuss several details that will be of importance in a real device design and experiments. In experiments, the easiest method to observe synchronization may be the spectral measurement of laser emissions. Since limit cycle oscillation frequencies are equivalent to laser oscillation frequencies, synchronization can be directly confirmed by the number of emission peaks in a measured spectrum. Namely, if a spectrum has a single emission peak, two lasers are synchronized, while if there are two emission peaks, they are not. Although coupling strengths between cavities are usually fixed in a device, it is still possible to actively tune the resonance frequency of a cold cavity^61–64,67^ and effectively change coupling strengths as shown in Fig. 2d. In this context, the spectral shape of laser emissions will be of interest. Below the lasing threshold, since laser cavities will behave as “cold cavities”, their emission spectrum is expected to exhibit normal-mode splitting. Meanwhile, above the lasing threshold the emission spectrum exhibits a single peak due to synchronization. Therefore, we may prove synchronization from the pump-power dependence of the change in spectral shape . Another promising experimental strategy to prove synchronization may be to pump two lasers independently and tune the respective laser frequencies by making use of the carrier-induced blue shift^49^. This strategy can be easily realized with spatially separated optical pumps or two electrodes for electric pumping.

The buried MQW PhC laser technique in the design of a real device is reported in Refs.^47,48^, where the PhC slab and buried PhC are composed of InP and InGaAsP/InGaAs, respectively. Furthermore, buried MQW PhC lasers can be pumped optically or electrically. If the photon lifetime of buried MQW PhC lasers is assumed to be $1/\gamma _1=1$ ps ($\sim$160 GHz), the frequency difference between two lasers corresponding to $\Delta \omega =0.01\gamma _1$, which is assumed in the simulations in Fig. 2, is $\Delta \omega =0.01\gamma _1\sim 1.6$ GHz. This laser frequency difference may seem to be severe for experimental realization (even with the state-of-the-art fabrication technology, the frequency difference between cavities may be about 50 GHz^68^), but we found that, qualitatively, the same synchronization can occur for a larger frequency difference. For example, synchronization with a laser frequency difference $\Delta \omega =0.1\gamma _1$ is discussed in Section 3 in the SM. Furthermore, we found that even if all the parameters of the three cavities including $\beta _{1,2}$ are moderately different, synchronization can occur (not shown).

## Conclusion and outlook

To conclude, we theoretically proposed a design of indirectly-coupled PhC cavity lasers that emulates the local Kuramoto model. In this study, we reinterpreted the injection-locking phenomenon of lasers as the synchronization of limit cycle oscillations. Furthermore, our design prevents laser oscillations from forming normal-modes (strong-coupling) with indirect coupling via additional cold cavities and realizes effective dissipative coupling without time-delay. Experimentally, this proposed structure will best be realized best by using buried MQW PhC cavities. First, after modelling laser oscillation with the Stuart-landau equation, we numerically demonstrated the synchronization of two indirectly-coupled PhC lasers using the coupled-mode equations of motion. Second, by applying the phase reduction theory to the two indirectly coupled lasers, we obtained corresponding phase equations of motion, which are equivalent to the local Kuramoto model. Finally, we briefly discussed synchronization dynamics for a one-dimensional chain of indirectly coupled PhC lasers and demonstrated that the proposed device can actually emulate the local Kuramoto chain.

For future perspectives, first of all, the one-dimensional local Kuramoto model briefly investigated in “Array configuration” section, already comprises rich physics that were actively investigated by detailed numerical simulations^46^ and renormalization group analysis^25,69^. Furthermore, very recently, Ref.^70^ demonstrated that even topological phenomena emerge in the one-dimensional chain of limit cycles. Thanks to the scalability of PhC cavities, the extension of the one-dimensional chain of PhC lasers to a two-dimensional array is straightforward, which is the realization of the celebrated two-dimensional local Kuramoto model^3,24–28,71^. Compared with the in-phase synchronization case, large-scale anti-phase synchronization has not yet been drawing attention. For instance, as Ref.^72^ indicates that a large anti-phase synchronization network is not possible, large-scale anti-phase synchronization itself may be of fundamental interest. Another important direction will be the inclusions of classical and quantum noise effects in indirectly coupled PhC lasers, which will provide spectral information. As we briefly discussed in “Discussion” section, we may prove synchronization in terms of the pump power dependence of spectral shape. In this direction, it is also easy to construct a quantum model corresponding to our coupled-mode equations. In fact, the quantum counterpart of the classical Stuart-Landau model is the Scully-lamb master equation^73,74^. Therefore, even the effect of quantum noises on synchronization^21,58,75^ may be tested with the proposed device. Moreover, since the synchronization problem in the local Kuramoto model is analogous to the energy minimization problem in the XY model, our device may be used for simulating the spin system in statistical physics^72,76^. Finally, from the standpoint of practical application, an injection-locked (synchronized) PhC laser array can be employed as a single-mode high-power PhC laser. Even though every PhC laser unavoidably has a different oscillation frequency, in the fully synchronized state, they behave as a laser with a single frequency. Furthermore, this type of a laser will also have high coherence because all laser phases are locked in the synchronized state.

## Methods

All the time evolutions were obtained by integrating the coupled-mode equations of motion with the conventional Runge-Kutta method. The synchronization trees were calculated as the mean oscillation frequencies of time evolutions. The calculation of the mean frequencies is based on the peak detection technique. To precisely determine the mean frequencies, long time evolutions (typically 6000$\gamma _1^{-1}$) were required.

## Supplementary Information

Supplementary Information
