# Supplementary material for: Emulating the local Kuramoto model with an injection-locked photonic crystal laser array
Source: Sci Rep. 2021 Apr 21;11:8587. doi: 10.1038/s41598-021-86982-w (PMC8060430; doi:10.1038/s41598-021-86982-w)
Supplement: Supplementary file 1 — Supplementary Information [file 41598_2021_86982_MOESM1_ESM.pdf]

Emulating the local Kuramoto model with an injection-locked photonic crystal laser array

N. Takemura,[1,2] K. Takata,[1,2] M. Takiguchi,[1,2] and M. Notomi[1,2,*]
[1]*Nanophotonics Center, NTT Corp., 3-1, Morinosato Wakamiya Atsugi, Kanagawa 243-0198, Japan*
[2]*NTT Basic Research Laboratories, NTT Corp., 3-1, Morinosato Wakamiya Atsugi, Kanagawa 243-0198, Japan*
(Dated: April 10, 2021)

1. DERIVATION OF PHASE EQUATIONS OF MOTION

We detail the calculations for the phase reduction analysis discussed in Section 4.2. Note that here we do not describe the phase reduction theory itself, which is explained, for example, in a review [1] and textbook [2]. First of all, we consider an orbit for laser L1 given by

$$\begin{pmatrix} x(\phi) \\ y(\phi) \end{pmatrix} = \sqrt{\frac{\epsilon_1}{\beta_1}} \begin{pmatrix} -\cos\phi \\ \sin\phi \end{pmatrix}, \tag{S1}$$

where $\phi = \omega_1 t$. Additionally, $x(\phi)$ and $y(\phi)$ represent real and imaginary parts of the laser field, respectively. Importantly, here, we consider the laser oscillation L1 given by Eq. (S1) as a "standard oscillator" for the phase reduction analysis. In the phase reduction theory, the function $\boldsymbol{Z}(\phi)$ called "sensitivity", which represents the response of limit cycle oscillation to perturbation, plays a central role. Fortunately, the analytical expression of the sensitivity $\boldsymbol{Z}(\phi)$ for the Stuart-Landau model [see Eq. (3) in the main text] is known [1] and, for the orbit given by Eq. (S1), is

$$\boldsymbol{Z}(\phi) = \begin{pmatrix} Z_x(\phi) \\ Z_y(\phi) \end{pmatrix} = \sqrt{\frac{\beta_1}{\epsilon_1}} \begin{pmatrix} \sin\phi \\ \cos\phi \end{pmatrix}. \tag{S2}$$

Our goal is to obtain the phase equations of motion in the form

$$\dot{\phi_1} = \omega_0 + \delta\Omega_1 + \Gamma_{12}(\phi_1 - \phi_2) \tag{S3}$$

$$\dot{\phi_2} = \omega_0 + \delta\Omega_2 + \Gamma_{21}(\phi_2 - \phi_1), \tag{S4}$$

where ϕ_1 and ϕ_2 are the phases of laser L1 and L2, respectively. Meanwhile, ω_0 is the oscillation frequency of the standard oscillator. Here, $\delta\omega_{1,2}$ represents a frequency shift originating from the difference between the standard oscillator and laser oscillation L1 and L2. Meanwhile, $\Gamma_{12}(\psi)$ and $\Gamma_{21}(\psi)$ are the phase coupling functions. We calculate $\delta\omega_{1,2}(\theta)$ and $\Gamma_{ij}(\psi)$ using the following formulae:

$$\delta\omega_{1,2} = \frac{1}{2\pi}\int_0^{2\pi} d\theta \boldsymbol{Z}(\theta)\cdot\delta\boldsymbol{F}_{1,2}(\theta) \tag{S5}$$

* E-mail: masaya.notomi.mn@hco.ntt.co.jp

and

$$\Gamma_{ij}(\psi) = \frac{1}{2\pi}\int_0^{2\pi} d\eta \boldsymbol{Z}(\eta+\psi)\cdot \boldsymbol{G}_{ij}(\eta), \tag{S6}$$

where $\delta\boldsymbol{F}_{1,2}(\theta)$ represents the difference between the standard oscillator and laser oscillation L1 and L2. Since, here, the standard oscillator is nothing else but laser L1, $\delta\boldsymbol{F}_1(\theta) = 0$ holds, and, consequently, we find that $\omega_0 = -\omega_1$ and $\delta\omega_1 = 0$. For the approximated equations of motion (10) and (11) in the main text, for the orbit Eq. (S1), the terms $\delta\boldsymbol{F}_2(\theta)$ and $\boldsymbol{G}_{12}(\eta)$ are represented as

$$\delta\boldsymbol{F}_2(\theta) = -\Delta\omega\sqrt{\frac{\epsilon_1}{\beta_1}}\begin{pmatrix}\sin\theta\\ \cos\theta\end{pmatrix} \tag{S7}$$

and

$$\boldsymbol{G}_{12}(\eta) = -\frac{2g_1g_2}{\Gamma_1}\sqrt{\frac{\epsilon_1}{\beta_1}}\begin{pmatrix}-\cos\eta\\ \sin\eta\end{pmatrix}. \tag{S8}$$

Now, Eqs (S5) and (S6) are easily calculated as

$$\delta\omega_2 = -\frac{1}{2\pi}\int_0^{2\pi} d\theta\Delta\omega(\sin^2\theta+\cos^2\theta) = -\Delta\omega = \omega_1-\omega_2 \tag{S9}$$

and

$$\begin{aligned}\Gamma_{12}(\psi) = \Gamma_{21}(\psi) &= \frac{2g_1g_2}{\Gamma_1}\frac{1}{2\pi}\int_0^{2\pi} d\eta\left\{\sin(\eta+\psi)\cos\eta - \cos(\eta+\psi)\sin\eta\right\}\\ &= \frac{2g_1g_2}{\Gamma_1}\frac{1}{2\pi}\int_0^{2\pi} d\eta\sin\psi = \frac{2g_1g_2}{\Gamma_1}\sin\psi.\end{aligned} \tag{S10}$$

Therefore, as discussed in the main text, if the adiabatic elimination approximation is valid, for the approximated coupled-mode equations, we obtain the corresponding phase equations of motion (17) and (18) in the main text.

2. SYNCHRONIZATION DYNAMICS WITH A LARGE COUPLING STRENGTH

Here, by simulating synchronization dynamics for two indirectly coupled lasers, we discuss the validity of the adiabatic elimination approximation when coupling strengths are larger than field decay rates. In the left panels of Fig. S1, in the same way as in Fig. 3(a) in the main text, we show the time evolutions of the fields $\mathrm{Re}[\alpha_{1,2}(t)]$ and $\mathrm{Re}[E_1(t)]$ calculated with the original equations [see Eqs. (6)-(8) in the main text] (solid lines) and calculated with the approximated equations [see Eqs. (10) and (11) in the main text] (dashed lines). Meanwhile, in the right panels of Fig. S1, we show the time evolutions of field intensities $|\alpha_{1,2}(t)|^2$ and $|E_1(t)|^2$ calculated with the original equations (solid lines) and calculated with the approximated equations (dashed lines). Importantly, in the same way as in Fig. 3(a) in the main text, since coupling is switched on for uncoupled laser oscillations at $t = 0$, Fig. S1 represents synchronization dynamics. Furthermore, to clearly show synchronization dynamics, we set the shifted frequencies as $\omega'_1 = 0.2\gamma_1$, $\omega'_2 = 0.21\gamma_1$, and $\Omega'_1 = 0.2\gamma_1$, which

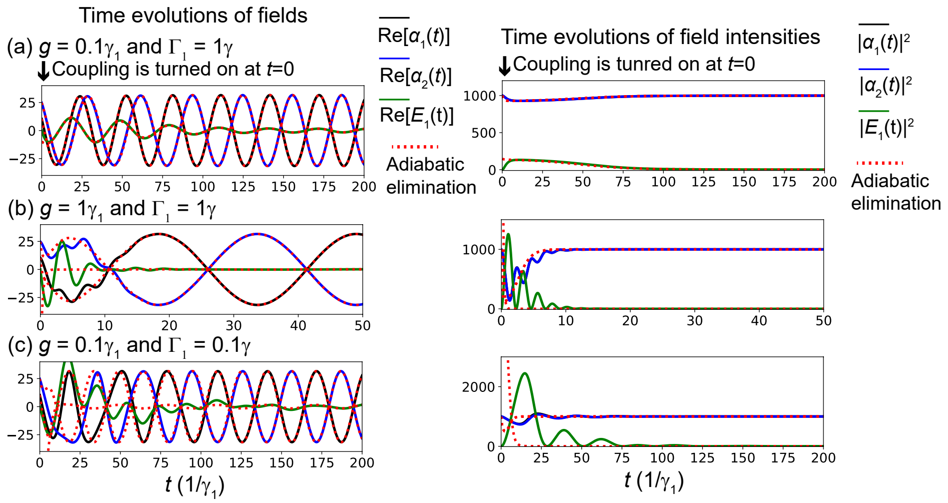

FIG. S1. Synchronization dynamics are simulated by turning on coupling at $t = 0$. The time evolutions of the fields $\mathrm{Re}[\alpha_{1,2}(t)]$ and $\mathrm{Re}[E(t)_1]$ are shown in the left panel, while field intensities $|\alpha_{1,2}(t)|^2$ and $\mathrm{Re}|E(t)_1|^2$ are shown in the right panel. The solid curves are simulations calculated with the original coupled-mode equations (6)-(8) in the main text, while the dashed curves are simulations obtained with the adiabatic elimination approximation [Eqs. (10) and (11) in the main text]. On the left panels, the shifted frequencies of the lasers are $\omega_1' = 0.2\gamma_1$ and $\omega_2' = 0.21\gamma_1$. The parameters used in (a) are the same as those in Fig. 3(a) in the main text. On the other hand, we used $g_1 = g_2 = 1\gamma_1$ and $\Gamma_1 = 1\gamma_1$ for (b), while we used $g_1 = g_2 = 0.1\gamma_1$ and $\Gamma_1 = 0.1\gamma_1$ for (c). The other parameters used in (b) and (c) are the same as those in Fig. 3(a) in the main text.

are the same as in Fig. 3(a).

For the all simulations in Fig. S1, we used the same parameters as in Fig. 3(a) in the main text except for the coupling strength $g_{1,2}$ and the decay rate of the cold cavity Γ_1. First, Fig. S1(a) shows the simulations with $g_1 = g_2 = 0.1\gamma_1$ and $\Gamma_1 = 1\gamma_1$. Therefore, the left panel in Fig. S1(a) is the same as Fig. 3(a) in the main text. Both the left and right panels in Fig. S1(a) indicate that the adiabatic elimination approximation perfectly reproduces synchronization dynamics calculated with the original coupled-mode equations [compare the solid and dashed curves in Fig. S1(a)]. Note that there is no oscillation in the field intensity dynamics in the right panel in S1(a). Second, for the simulation in Fig. S1(b), we increased the coupling strength as $g_{1,2} = 1\gamma_1$, while we fixed the decay rate of the cold-cavity as $\Gamma_1 = 1\gamma_1$. As the left panel in Fig. S1(b) indicates, the adiabatic elimination approximation fails to reproduce the turn-on dynamics (compare the solid and dashed curves until $t \simeq 10\gamma_1^{-1}$). Meanwhile, the adiabatic elimination approximation succeeds in reproducing the "steady-state" synchronized laser oscillations (compare the solid and dashed curves after $t \simeq 10\gamma_1^{-1}$). The right panel in Fig. S1(b) indicates that the failure of the adiabatic elimination approximation is associated with the coherent oscillations of field intensities between the cavities in the turn-on dynamics, which originate from the (near) strong-coupling condition. Furthermore, as Fig. S1(b) shows, it is when the coherent intensity oscillations are damped and the system reaches the "steady-state" around $t \simeq 10\gamma_1^{-1}$ that the adiabatic elimination approximation becomes valid. Finally, in Fig. S1(c), we show simulations with the smaller decay rate of the cold cavity $\Gamma_1 = 0.1\gamma_1$, but with a fixed coupling strength $g_{1,2} = 0.1\gamma_1$. As in

Fig. S1(b), the left panel in Fig. S1(c) indicates that the adiabatic elimination approximation cannot reproduce the turn-on dynamics (before $t \simeq 100\gamma_1$), while the approximation starts to well approximate the "steady-state" synchronized laser oscillations after $t \simeq 100\gamma_1$. This result can again be explained in terms of the coherent oscillation of the field intensities originating from the (near) strong coupling condition [see the right panel in Fig. S1(c)].

In summary, we found that when coherent intensity oscillation is present in the strong coupling regime, the adiabatic elimination approximation fails to reproduce turn-on dynamics. Intuitively, since the adiabatic elimination approximation assumes a large time-scale difference between the field dynamics in the cold cavity (fast dynamics) and in the laser cavities (slow dynamics), when these fields exhibit coherent oscillations, the time-scale separation becomes impossible and, consequently, the adiabatic elimination approximation breaks down. Therefore, the strict conditions required for the adiabatic elimination approximation are $\omega_1 \simeq \omega_2 \simeq \Omega_1$ and $g_{1,2} < \gamma_{1,2}, \Gamma_1$ [see Eq. (12) in the main text]. We also stress that even when the above conditions are not satisfied, the stable synchronization of laser oscillations itself can be realized, and the adiabatic elimination approximation well describes "steady-state" synchronized laser oscillations as we find in Fig.S1(b) and (c), which is because the coherent intensity oscillation is damped in the "steady-state" and the time-scale separation becomes possible.

3. SYNCHRONIZATION OF LASERS WITH A LARGER OSCILLATION FREQUENCY DIFFERENCE

Here, we discuss the synchronization of two indirectly coupled lasers with a frequency difference $\Delta\omega \equiv \omega_2 - \omega_1 = 0.1\gamma_1$, which is ten times larger than that used in Section 3 in the main text. In fact, all the parameters except for ω_2 and $g_{1,2}$ are the same as those used in Section 3. Therefore, the shifted resonance frequencies of the laser cavities and cold cavities are set as $\omega'_1 = 1\gamma_1$, $\omega'_2 = 1.1\gamma_1$, and $\Omega'_1 = 1\gamma_1$. First, by simulating the coupled-mode equations (6)-(8) in the main text, we obtained the synchronization tree in the left panel in Fig. S2, where the average frequency of laser oscillation $\bar{\omega}'_{1,2}$ is shown as a function of $g_{1,2}$. The left panel in Fig. S2 indicates that synchronization can occur even with this laser frequency difference $\Delta\omega = 0.1\gamma_1$. However, comparing Fig. S2 with Fig. 2(c) in the main text, we can easily find that the synchronization is slightly asymmetric between $\bar{\omega}'_1$ and $\bar{\omega}'_2$, which is because there is a relatively large asymmetry in the resonance frequencies of the cavities as $\omega'_1 = 1\gamma_1$, $\omega'_2 = 1.1\gamma_1$, and $\Omega'_1 = 1\gamma_1$.

Second, in the right panel in Fig. S2, the solid black and blue curves are the same as those in the left panel, but the red dashed curves are calculated with the corresponding phase equations of motion (16) and (17) in the main text. The corresponding phase equations of motion can qualitatively reproduce the original synchronization tree (compare the solid and dashed curves), the two synchronization trees do not perfectly coincide with each other. In fact, the ideal synchronization tree obtained with the phase equations of motion (see the dashed curves) cannot reproduce the asymmetry between $\bar{\omega}'_1$ and $\bar{\omega}'_2$ in the solid curves. This difference between the two approaches originates from the fact that the adiabatic elimination approximation partly fails because the adiabatic elimination condition $\omega_1 \simeq \omega_2 \simeq \Omega_1$ [see Eq. (12) in the main text] is partly violated, which results in decreasing the approximation of the phase equations of motion.

Finally, we comment on the critical coupling strengths of synchronization, which is indicated both by the solid and dashed curves as $g_1 = g_2 = \sqrt{0.025}\gamma_1$. We show that the

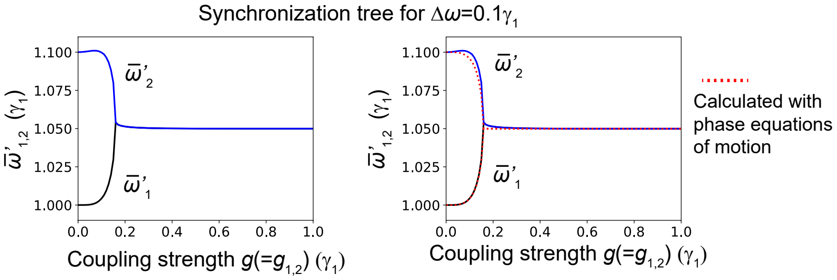

FIG. S2. Mean frequency of the oscillation frequency $\bar{\omega}_{1,2}$ of the laser L1 and L2 as a function of the coupled strength $g_{1,2}$. The solid black and blue curves in the left and right panels represent the synchronization trees calculated with the coupled-mode equations (6)-(8) in the main text. Meanwhile, the red dashed curves are the synchronization trees calculated with the corresponding phase equations of motion (16) and (17) in the main text.

critical coupling strengths agree with the prediction of the phase equation of motion (15) in the main text. Namely, for $\Gamma_1 = 1\gamma_1$ and $\Delta\omega = 0.1\gamma_1$, when $g_1 = g_2 = \sqrt{0.5}\gamma_1$, the synchronization condition $-4g_1g_2/\Gamma_1 \leq \Delta\omega \leq 4g_1g_2/\Gamma_1$ is satisfied. We also found that with a further increase in the coupling strength $g_{1,2}$, the adiabatic elimination approximation becomes better around $g_{1,2} = 0.2\gamma_1$ and that both solid and dashed synchronization trees reach $\bar{\omega}'_{1,2} \to 1.05\gamma_1$, which is the mean frequency for $\omega'_1 = 1\gamma_1$ and $\omega'_2 = 1.1\gamma_1$ without coupling.

4. SYNCHRONIZATION OF CLASS-B LASERS

In this section, we demonstrate that all the arguments in the manuscript based on the Stuart-Landau equation can be reproduced even quantitatively with class-B lasers where $\gamma_c > \gamma_{\|}$ [see Eqs. (1) and (2) in the main text]. Since the adiabatic elimination of the carrier degree of freedom cannot be allowed, we need to directly simulate the following coupled-mode equations for two indirectly coupled class-B lasers:

$$\dot{\alpha}_1 = -i\omega_1\alpha_1 - \frac{1}{2}\gamma_1\alpha_1 + \frac{1}{2}\beta_1\tilde{\gamma}_1 N_1\alpha_1 - ig_1E_1 \tag{S11}$$

$$\dot{N}_1 = -\tilde{\gamma}_1 N_1 - \beta\tilde{\gamma}_1 N_1|\alpha_1|^2 + P_1 \tag{S12}$$

$$\dot{E}_1 = -i\Omega_1 E_1 - \frac{1}{2}\Gamma_1 E_1 - ig_1\alpha_1 - ig_2\alpha_2 \tag{S13}$$

$$\dot{\alpha}_2 = -i\omega_2\alpha_2 - \frac{1}{2}\gamma_2\alpha_2 + \frac{1}{2}\beta_2\tilde{\gamma}_2 N_2\alpha_2 - ig_2E_1 \tag{S14}$$

$$\dot{N}_2 = -\tilde{\gamma}_2 N_2 - \beta_2\tilde{\gamma}_2 N_2|\alpha_2|^2 + P_2, \tag{S15}$$

where $N_{1,2}$ represents the carrier number for laser L1,2, while $\tilde{\gamma}_{1,2}$ is the decay rate of the carrier $N_{1,2}$. With Eq. (4) in the main text, the pump power P_i is connected with the pump parameter ϵ_i as

$$P_i = \frac{\gamma_i}{\beta_i}(1+\epsilon_i). \tag{S16}$$

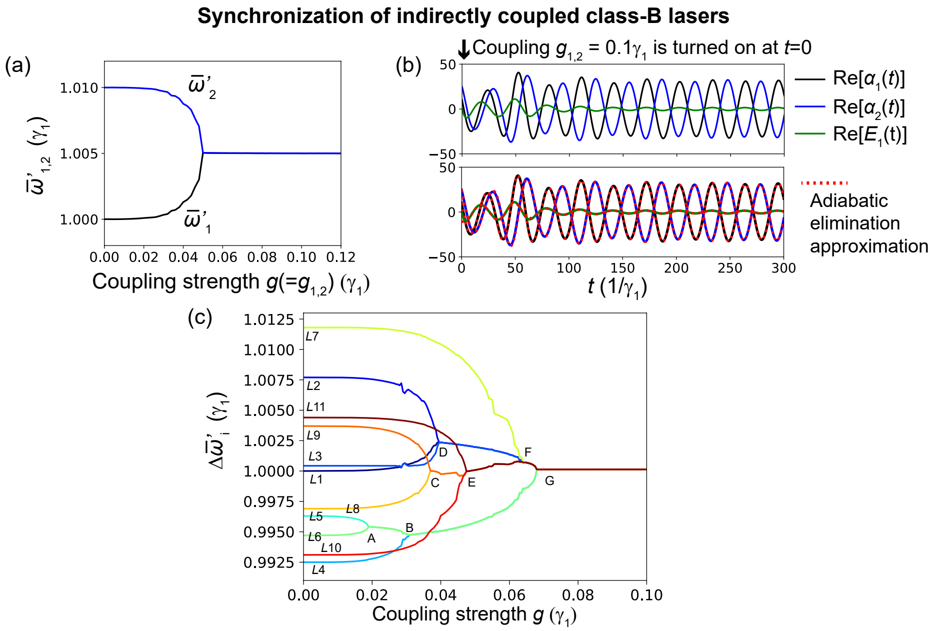

FIG. S3. (a) Mean frequencies of two indirectly coupled class-B lasers $\bar{\omega}_1$ and $\bar{\omega}_2$ as a function of coupled strength $g_{1,2}$. (b) Synchronization dynamics of two indirectly coupled class-B lasers, where coupling ($g_{1,2} = 0.1\gamma_1$) is turned on at $t = 0$. Time evolution of the laser fields $\mathrm{Re}[\alpha_{1,2}(t)]$ and $\mathrm{Re}[E(t)_1]$ are shown. The upper panel shows the time evolutions calculated with the original coupled-mode equations (S14)-(S15). In the lower panel, the time evolutions calculated with the adiabatic elimination approximation Eqs. (S17)-(S20) are plotted as red dashed curves on the original curves. The shifted frequencies of the laser and cold cavities $\omega_1' = 0.2\gamma_1$, $\omega_2' = 0.21\gamma_1$, and $\Omega_1' = 0.2\gamma_1$ are used. For the other parameters in (a) and (b), we used the same values as in Fig. 2(c) and 3(a) in the main text except for the carrier lifetimes $\tilde{\gamma}_2 = \tilde{\gamma}_1 = 0.01\gamma_1$. (c) The mean oscillation frequencies of the eleven lasers $\bar{\omega}_i'$ as a function the coupling strength $g_i = g$ for all i. The synchronization points are denoted by A-G. The parameter values other than $\tilde{\gamma}_i = 0.01\gamma_1$ are the same as those used in Fig. 4(b) in the main text.

The definitions of the other parameters are the same as in Eqs (6)-(8) in the main text. For all simulations in this supplemental material, the photon and carrier lifetimes are set as $\tilde{\gamma}_1 = \tilde{\gamma}_2 = 0.01\gamma_1$ and $\gamma_2 = \gamma_1$, which is clearly the class-B regime. For the other parameters, we use the same values as in the main text. Namely, we use $\beta_1 = \beta_2 = 0.001$ and $\varepsilon_1 = \varepsilon_2 = 1.0$. For the frequencies of the laser and cold cavity, we use the shifted frequencies $\omega_1' = 1\gamma_1$, $\omega_2' = 1.01\gamma_1$, and $\Omega_1' = 1\gamma_1$. First, in Fig. S3(a), we show the mean frequencies of the two lasers $\bar{\omega}_1'$ and $\bar{\omega}_2'$ as a function of the coupling strength between cavities $g_{1,2}$. We found that Fig. S3(a) is even quantitatively the same as Fig. 2(c) in the main text.

The only difference between the class-A and class-B lasers is the response to amplitude perturbation. In the same way as in Fig. 3(a) in the main text, synchronization dynamics are shown in Fig. S3(b), where coupling ($g_{1,2} = 1\gamma_1$) is turned on at $t = 0$. The turn-on of coupling works as perturbation and induces relaxation oscillations in the synchronization dynamics [see the oscillations of the amplitudes in Fig. S3(b)], which is the characteristic of class-B lasers. However, after the relaxation oscillations are damped, the two class-B

laser oscillations exhibit anti-phase synchronization, which is the same as class-A lasers. Additionally, in the lower panel in Fig. S3(b), the red dashed curves represent approximated equations of motion

$$\dot{\alpha}_1 = -i\omega_1\alpha_1 - \frac{1}{2}\gamma_1\alpha_1 - \frac{2g_1^2}{\Gamma_1}\alpha_1 + \frac{1}{2}\beta_1\tilde{\gamma}_1 N_1\alpha_1 - \frac{2g_1g_2}{\Gamma_1}\alpha_2 \tag{S17}$$

$$\dot{N}_1 = -\tilde{\gamma}_1 N_1 - \beta\tilde{\gamma}_1 N_1|\alpha_1|^2 + P_1 \tag{S18}$$

$$\dot{\alpha}_2 = -i\omega_2\alpha_2 - \frac{1}{2}\gamma_2\alpha_2 - \frac{2g_2^2}{\Gamma_1}\alpha_2 + \frac{1}{2}\beta_2\tilde{\gamma}_2 N_2\alpha_2 - \frac{2g_1g_2}{\Gamma_1}\alpha_1 \tag{S19}$$

$$\dot{N}_2 = -\tilde{\gamma}_2 N_2 - \beta_2\tilde{\gamma}_2 N_2|\alpha_2|^2 + P_2, \tag{S20}$$

which are obtained by adiabatically eliminating the field of the cold-cavity E_1 with Eq. (9) in the main text. The lower panel in Fig. S3(b) clearly indicates that the adiabatic elimination of the field E_1 is a very good approximation even for class-B lasers. By applying the numerical phase reduction to the rate equations of motion (1) and (2) in the main text[1, 3], we found that the sensitivity $\boldsymbol{Z}(\phi)$ (see Section 1 in this supplemental material) is given by $\boldsymbol{Z}(\phi) = (Z_x(\phi), Z_y(\phi)), Z_N(\phi)) = \sqrt{\beta_1/\epsilon_1}(-\cos\phi, \sin\phi, 0)$. Thus, the phase equations of motion corresponding to Eqs. (S17)-(S20) are the same as Eqs (17) and (18) in the main text. Accordingly, the antisymmetric part of the phase coupling function is also given by $\Gamma_a(\psi) = (4g_1g_2/\Gamma_1)\sin\psi$, and phase locking occurs at the phase $\psi = \phi_2 - \phi_1 = \pi$ (anti-phase synchronization). Since the phase equations of motion are the same between class-A and class-B lasers, all the arguments on phase dynamics for class-A lasers can be applied to class-B lasers.

Finally, in Fig. S3(c), we show a synchronization tree for eleven indirectly coupled class-B lasers. All the parameters except for carrier lifetime $\tilde{\gamma}_i = 0.01\gamma_1$, which is not present in the main text, are the same as those in Fig. 4(b) in the main text. Figure S3(c) is again quantitatively the same as the synchronization tree shown in Fig. 4(b) in the main text.

5. IMPACT OF THE LINEWIDTH ENHANCEMENT FACTOR ON SYNCHRONIZATION

In this section, we briefly discuss the impacts of the linewidth enhancement factor (the Henry factor [4]) α_{H} on synchronization of indirectly coupled lasers. Since the linewidth enhancement factor is not negligible in semiconductor lasers, its effects will be very important for real experiments using PhC lasers. In the same way as Refs. [4–6], we introduce the linewidth enhancement factor α_{H} into rate equations (1) and (2) in the main text as

$$\dot{\alpha} = -i\omega_c\alpha - \frac{1}{2}\gamma_c\alpha + (1 - i\alpha_{\mathrm{H}})\frac{1}{2}\beta\gamma_{\|}N\alpha \tag{S21}$$

$$\dot{N} = -\gamma_{\|}N - \beta\gamma_{\|}N|\alpha|^2 + P. \tag{S22}$$

The above modified rate equations indicate that the factor α_{H} contributes to a carrier-induced blue shift. In this section, we set the value of the linewidth enhancement factor $\alpha_{\mathrm{H}} = 4.0$, which is the measured value for buried multiple quantum well PhC lasers [7]. For indirectly coupled lasers coupled-mode equations including the linewidth enhancement effect

are explicitly written as

$$\dot{\alpha}_1 = -i\omega_1\alpha_1 - \frac{1}{2}\gamma_1\alpha_1 + (1 - i\alpha_{\rm H})\frac{1}{2}\beta_1\tilde{\gamma}_1 N_1\alpha_1 - ig_1 E_1 \tag{S23}$$

$$\dot{N}_1 = -\tilde{\gamma}_1 N_1 - \beta\tilde{\gamma}_1 N_1|\alpha_1|^2 + P_1 \tag{S24}$$

$$\dot{E}_1 = -i\Omega_1 E_1 - \frac{1}{2}\Gamma_1 E_1 - ig_1\alpha_1 - ig_2\alpha_2 \tag{S25}$$

$$\dot{\alpha}_2 = -i\omega_2\alpha_2 - \frac{1}{2}\gamma_2\alpha_2 + (1 - i\alpha_{\rm H})\frac{1}{2}\beta_2\tilde{\gamma}_2 N_2\alpha_2 - ig_2 E_1 \tag{S26}$$

$$\dot{N}_2 = -\tilde{\gamma}_2 N_2 - \beta_2\tilde{\gamma}_2 N_2|\alpha_2|^2 + P_2, \tag{S27}$$

where all the parameters other than $\alpha_{\rm H}$ are already defined in Section 4 in this supplemental material.

Figure S4(a) shows the time evolutions of two indirectly coupled lasers calculated with Eqs. (S23)-(S27). For the parameters, we use the carrier lifetime $\tilde{\gamma}_1 = \tilde{\gamma}_2 = 0.01\gamma_1$, linewidth enhancement factor $\alpha_{\rm H} = 4.0$, and $\Omega'_1 = 3\gamma_1$. The other parameters are the same values as in Fig. 2(a) in the main text: $\omega'_1 = 1\gamma_1$, $\omega'_2 = 1.01\gamma_1$, $\beta_1 = \beta_2 = 0.001$, $\varepsilon_2 = \varepsilon_1 = 1.0$, and $\gamma_2 = \gamma_1 \equiv 1$. Later, we explain the reason why the frequency of the cold cavity is very different from those of laser cavities ($\Omega'_1 = 3\gamma_1$), which actually plays a key role in the synchronization of lasers with the linewidth enhancement factor . First, we discuss how the factor $\alpha_{\rm H}$ modifies independent laser oscillations . The upper panel in Fig. S4(a) represents the time evolutions of the real parts of the fields Re$[\alpha_1(t)]$ and Re$[\alpha_2(t)]$ without coupling $g_{1,2} = 0$. We notice that the laser oscillation frequencies are much higher than those in Fig. 2(a) in the main text. In fact, the laser oscillation frequencies are found to be $\bar{\omega}'_1 = 3\gamma_1$ and $\bar{\omega}'_2 = 3.01\gamma_1$ for lasers L1 and L2, respectively. The increases in the laser oscillation frequencies originate from the carrier-induced blue shift associated with $\alpha_{\rm H}$. With $\alpha_{\rm H}$, the oscillation frequency of a laser is shifted as $\omega_c + \alpha_H\beta\gamma_{\|}N/2$. Since the saturated carrier number above the lasing threshold is $N = \gamma_c/(\beta\gamma_{\|})$, the frequency shift of laser oscillation is $\alpha\gamma_c/2$. In our case, the laser frequency shift is estimated as $\alpha\gamma_c/2 = 2\gamma_1$, which coincides with the simulation. Second, we introduce coupling $g_{1,2} = 0.2\gamma_1$ in the lower panel of Fig. S4(a), which shows that anti-phase synchronization can occur even with the presence of the linewidth enhancement factor $\alpha_{\rm H} = 4.0$.

Now, we show the mean frequencies of the two lasers $\bar{\omega}'_1$ and $\bar{\omega}'_2$ as a function of the coupling strength $g_{1,2}$ in Fig. S3(b). In stark contrast to Fig. 2(c) in the main text, the synchronization tree shown in Fig. S3(b) is asymmetric, which originates from the modulations of laser frequencies induced by the changes in the carrier numbers. Although the changes in the carrier numbers are present even in Fig. 2(c) in the main text, they do not contribute to frequency shifts because $\alpha_{\rm H} = 0$ in the main text. Figure 2(b) indicates that synchronization occurs at $g_{1,2} = 0.056\gamma_1$ and, with a further increase in the coupling strength, the synchronized laser frequencies gradually decrease to $\bar{\omega}'_1 = \bar{\omega}'_2 = 3.005\gamma_1$, which is the mean frequency of the two uncoupled lasers.

Now, we explain the importance of the cold-cavity frequency for synchronization of lasers with the linewidth enhancement factor. In Fig. S4, we set the frequency of the cold cavity as $\Omega_1 = 3\gamma_1$ to compensate the carrier-induced blue shift induced by the factor $\alpha_{\rm H}$. With this trick, the adiabatic elimination condition for cavity frequencies [see Eq. (12) in the main text] effectively holds as $\omega_1 + 2\gamma_1 \simeq \omega_2 + 2\gamma_1 \simeq \Omega_1$, and the adiabatic elimination of the cold cavity field leads to effective dissipative coupling. To check the validity of the adiabatic elimination approximation, we use Fig. S4(c) and (d). Like Fig. 3(a) in the

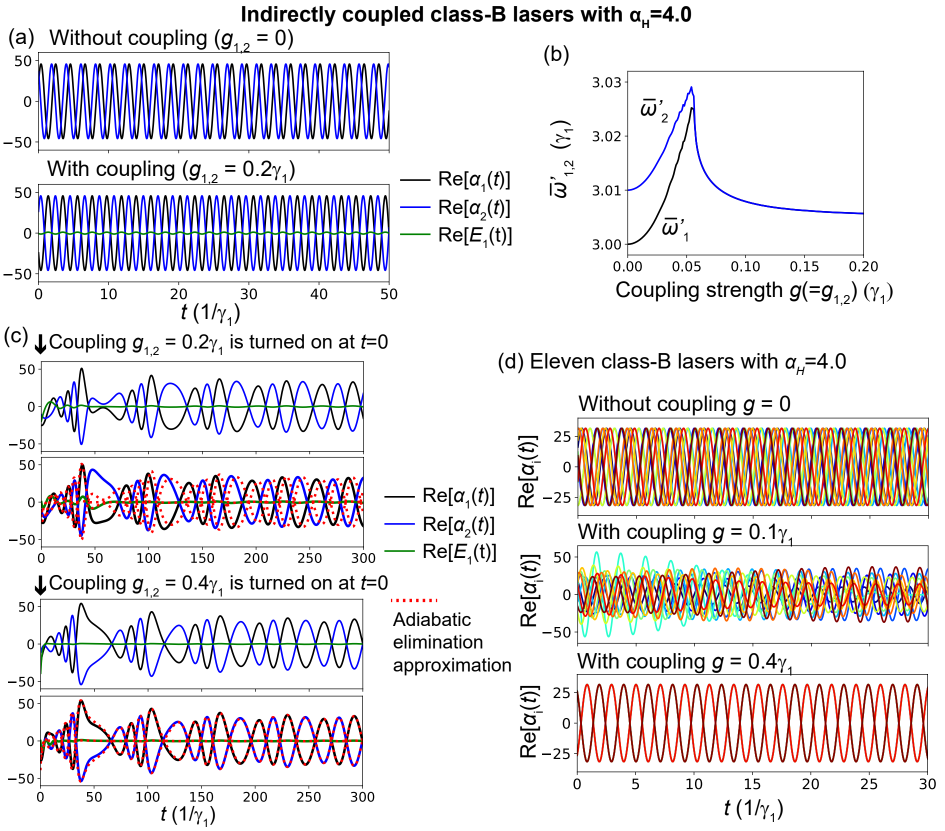

FIG. S4. Simulations with the linewidth enhancement parameter $\alpha_{\rm H} = 4.0$. (a) Time evolutions of the real part of the field $\text{Re}[\alpha_{1,2}(t)]$ without $g_{1,2} = 0$ and with coupling $g_{1,2} = 0.2\gamma_1$. The shifted cavity frequencies are $\omega_1' = 1\gamma_1$ and $\omega_2' = 1.01\gamma_1$, and $\Omega_1' = 3\gamma_1$. (b) Mean frequency of the laser oscillation $\bar{\omega}_{1,2}$ as a function of coupled strength $g_{1,2}$. (c) Synchronization dynamics of the fields $\text{Re}[\alpha_{1,2}(t)]$ and $\text{Re}[E(t)_1]$. Coupling ($g_{1,2} = 0.2\gamma_1$ and $0.4\gamma_1$) is turned on at $t = 0$. The solid and dashed curves represent synchronization dynamics calculated with the original and approximated equations of motion, respectively. Here, the cavity frequencies are shifted as $\omega_1' = -1.8\gamma_1$ and $\omega_2' = -1.79\gamma_1$, and $\Omega_1' = 0.2\gamma_1$. For the other parameters in (a), (b), and (c), we used $\beta_2 = \beta_1 = 0.001$, $\varepsilon_2 = \varepsilon_1 = 1.0$, $\gamma_2 = \gamma_1 \equiv 1$, $\tilde{\gamma}_2 = \tilde{\gamma}_1 = 0.01\gamma_1$, and $\Gamma_1 = 1\gamma_1$. (d) Time evolutions of the real parts of the eleven indirectly coupled laser fields for $g(= g_i) = 0$ (top), $0.1\gamma_1$ (middle), and $0.4\gamma_1$ (bottom). The parameter values except for $\tilde{\gamma}_i = 0.01\gamma_1$, $\alpha_{\rm H} = 4.0$, and $\Omega_i = 3\gamma_1$ are the same as those used in Fig. 4(b) in the main text.

main text, Fig. S4(c) and (d) show synchronization dynamics calculated with the original equations of motion (S23)-(S27) (black and blue solid curves) and approximated equations of motion obtained with the adiabatic elimination of the field E_1 (red dashed curves). When the coupling strength is $g_{1,2} = 0.2\gamma_1$, the approximated equations of motion can reproduce the frequencies of lasers but cannot reproduce their phases. Meanwhile, when the coupling strength is further increased to $g_{1,2} = 0.4\gamma_1$, the approximated equations of motion can reproduce both frequencies and phases of the lasers. We also note that if the frequency of the cold cavity remains as $\Omega_1 = 1\gamma_1$, the adiabatic elimination fails and the laser oscillation

becomes chaotic for a certain range of $g_{1,2}$ (not shown). This chaos emission of injection-locked lasers induced by $\alpha_{\rm H}$ is studied in Refs. [5, 8, 9]. Although the laser chaos is beyond the scope of this paper, this regime will be of great interest because our proposed device can also be used as an on-chip chaotic light emitter.

Finally, we demonstrate that large-scale synchronization can be possible even with a non-negligible linewidth enhancement factor. The time evolutions of eleven indirectly coupled lasers with linewidth enhancement factors are shown in Fig. S4(d), where we used the carrier lifetime $\tilde{\gamma}_i = 0.01\gamma_1$ and $\Omega_i = 3\gamma_1$, while all the laser cavities have $\alpha_{\rm H} = 4.0$. The other parameters are the same as those in Fig. 4(b) in the main text. The upper, middle, lower panels in Fig. S4(d) represent the time evolutions for $g_i = 0$, $0.1\gamma_1$, and $0.4\gamma_1$, respectively. Note that, with $\alpha_{\rm H} = 4.0$, unfortunately, we cannot show a synchronization tree like the one in Fig. 4(b) in the main text. This is because chaotic laser oscillations emerge for a certain coupling strength [for example, see $g = 0.1\gamma_1$ in Fig. 4(d)]. On the other hand, when the coupling strength reaches a threshold, fully anti-phase synchronized oscillations can be realized even with $\alpha_{\rm H} = 4.0$ [see $g = 0.4\gamma_1$ in Fig. S4(d)], which is an important indication for real experiments. Qualitatively, we may summarize the behavior of indirectly coupled laser with $\alpha_{\rm H} = 4.0$ as follows. First, when non-zero coupling is introduced, laser oscillations are quasiperiodic or chaotic. It is not clear whether or not there is a threshold coupling strength of chaos transition as in Ref. [5]. Second, as coupling strength is increased, the chaotic behavior is enhanced as shown in Fig. 4(d) for $g = 0.1\gamma_1$. Finally, as the coupling strength is further increased, the chaotic laser oscillations suddenly exhibit anti-phase synchronization, which is the synchronization transition. For the parameters used in Fig. 4(d), the synchronization transition occur around $g(= g_i) = 0.395\gamma_1$.

6. LARGE-SCALE SYNCHRONIZATION WITH DISORDERED PARAMETERS

Here, we discuss the large-scale synchronization of eleven indirectly coupled lasers but with disordered parameters. In Fig. 4(b) in the main text, for simplicity, we assumed that all cavities have the same parameter values except for laser cavity frequencies. However, this assumption is unrealistic because the parameters of all laser and cold cavities unavoidably have different values. Therefore, it is important to show the possibility of the large-scale synchronization of indirectly coupled lasers with disordered parameters. Note that, in this section, our objective is not to quantitatively investigate the upper limit of the disorder of parameters for synchronization, but is only to demonstrate that large-scale synchronization can be possible even when parameter values are not the same.

We simulate eleven indirectly coupled lasers with the same configuration as in Fig. 4(a) in the main text, but all parameters except for coupling strengths g_i have slightly different values. The parameters of all laser (ϵ_i, β_i, and γ_i) and cold cavities (Ω_i and Γ_i) are randomly distributed around their mean values, which is summarized in Table S1. Note that the laser cavities have the same frequencies as those in Fig. 4 in the main text. First, in Fig. S5(a), we show the mean frequencies of the eleven lasers as a function of the coupling strength $g(= g_i)$. The indices of synchronization points A-G are denoted in the same way as in Fig. 4(b) in the main text. The synchronization tree shown in Fig. S5(a) well resembles that in Fig. 4(b) in the main text and clearly indicates that large-scale synchronization can be realized even when the cavities do not have equal parameter values. However, synchronization behavior around synchronization point F is more complicated than that in Fig. 4(b) in the main text. Interestingly, de-synchronization, discussed in Ref. [10], may be observed around point F.

TABLE S1. Parameter values for eleven laser and ten cold cavities

Index i	ω_i' $[\gamma_1]$	γ_i $[\gamma_1]$	β_i	ϵ_i	Ω_i $[\gamma_1]$	Γ_i $[\gamma_1]$
1	1.0000	1	0.0011	1.022	0.9906	0.9968
2	1.0077	1.12	0.0012	0.923	0.9988	1.0068
3	1.0004	1.11	0.00098	0.89	1.0011	1.0011
4	0.9925	1.025	0.00099	1.035	0.9965	1.0035
5	0.9963	0.962	0.0011	0.99	1.0220	0.9882
6	0.9947	0.9977	0.00096	1.053	1.0032	1.0053
7	1.0118	0.912	0.0012	0.979	0.9983	0.9979
8	0.9969	1.09	0.0013	1.061	0.9974	1.0061
9	1.0037	0.999	0.0012	0.983	1.0072	0.9983
10	0.9931	1.0076	0.00095	0.951	0.9901	1.0049
11	1.0044	1.033	0.00096	1.036		

Second, in Fig. S5(b), we show the time evolutions of the laser oscillations for $g = 0$, $0.1\gamma_1$, and $0.2\gamma_1$ in the top, middle, and bottom panels of Fig. S5(b), respectively. Due to the difference in β_i and ϵ_i, Fig. S5(b) indicates that the amplitudes of all the laser oscillations are slightly different. As we expect, synchronization occurs with $g = 0.1\gamma_1$ [see the middle panel Fig. S5(b)], but the pair of synchronized oscillations have slightly different phases. When the coupling is increased to $g = 0.2\gamma_1$, all the pairs of synchronized oscillations have the same phase [see the bottom panel Fig. S5(b)].

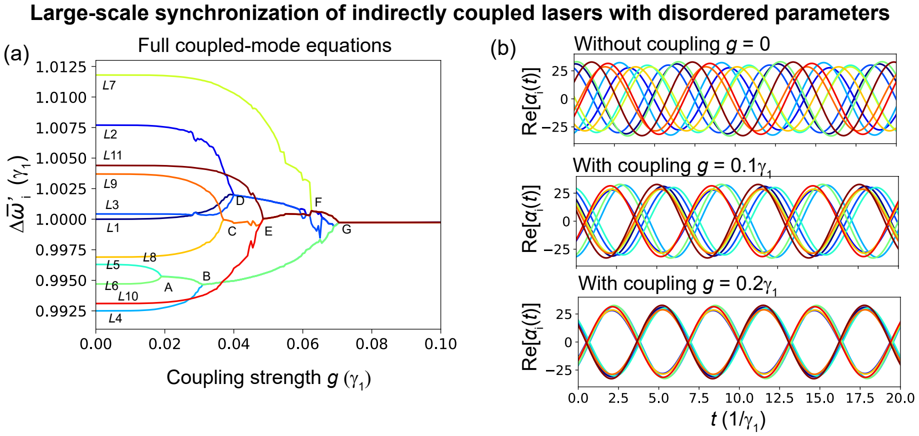

FIG. S5. Simulations for eleven indirectly coupled lasers with disordered parameters. The parameter values of the laser and cold cavities are summarized in Table S1. (a) The mean oscillation frequencies of the eleven lasers $\bar{\omega}_i'$ are shown as a function the coupling strength $g_i = g$ for all i. The synchronization points are denoted by A-G in the same way as in Fig. 4(b) in the main text. (b) Time evolutions of the real parts of the fields in all the laser cavities for $g = 0$ (top), $0.1\gamma_1$ (middle), and $0.2\gamma_1$ (bottom).

[1] H. Nakao, Contemporary Physics **57**, 188 (2016), https://doi.org/10.1080/00107514.2015.1094987.

[2] Y. Kuramoto, *Chemical oscillations, waves, and turbulence* (Courier Corporation, 2003).
[3] N. Takemura, M. Takiguchi, and M. Notomi, Opt. Express **28**, 27657 (2020).
[4] C. Henry, IEEE Journal of Quantum Electronics **18**, 259 (1982).
[5] H. G. Winful and S. S. Wang, Applied Physics Letters **53**, 1894 (1988), https://doi.org/10.1063/1.100363.
[6] P. Hamel, S. Haddadi, F. Raineri, P. Monnier, G. Beaudoin, I. Sagnes, A. Levenson, and A. M. Yacomotti, Nature Photonics **9**, 311 (2015).
[7] J. Kim, A. Shinya, K. Nozaki, H. Taniyama, C.-H. Chen, T. Sato, S. Matsuo, and M. Notomi, Opt. Express **20**, 11643 (2012).
[8] S. S. Wang and H. G. Winful, Applied Physics Letters **52**, 1774 (1988), https://doi.org/10.1063/1.99622.
[9] S. Hwang and J. Liu, Optics Communications **183**, 195 (2000).
[10] Z. Zheng, G. Hu, and B. Hu, Phys. Rev. Lett. **81**, 5318 (1998).
